# Retraction: Anti-cancer effects of polyphenol-rich sugarcane extract

**DOI:** 10.1371/journal.pone.0343944

**Published:** 2026-03-04

**Authors:** The PLOS One Editors

After this article [1] was published, concerns were raised about Figs 2, 3, and 6.

Specifically:

- In Fig 2, the SKOV-3 cells (ovarian) (-) and the CT26 cells (colon) (-) panels appear similar.
- Fig 3 appears to report the results of statistical analyses, despite the individual-level data for this article available via Figshare [2] appearing to report only two repeats/replicates.
- In Fig 6, the Untreated, PRSE, and Ibuprofen panels for the LIM2045 cells appear more similar than would be expected from independent samples.
- The Results and Discussion sections refer to the results in Fig 6 as showing significant increases, decreases, or differences, but no statistical tests are reported.

Regarding Fig 2, the first author stated that an error occurred during figure preparation and that the SKOV-3 cells (ovarian) (-) panel is incorrect. The first author agreed that the Fig 6 Untreated, PRSE, and Ibuprofen panels for the LIM2045 cells appear similar and provided the original FACS data files for these panels. Upon editorial review, these data did not resolve the above concerns for Fig 6 and raised additional concerns that the Fig 6 results appear to rely on a single biological sample without replication.

The first author stated that Figs 3 and 5 report two technical replicates and that these assays were performed only once. They stated that Fig 4 presents a single representative result from a flow cytometry experiment. Upon further editorial review of the underlying individual-level data provided by the authors, the values and reported changes did not correspond to the results presented in Fig 4.

The *PLOS One* Editors consider the number of replicates in Fig 3 insufficient for statistical analyses, and that the lack of replication of the results reported in Figs 4 and 6 does not adequately support the conclusions drawn from these figures. As such, this article does not meet the journal’s third publication criterion [3].

During the editorial reassessment of this article [1], PLOS identified concerns regarding the article’s peer review. The Editors have no evidence of any author involvement in the peer review concerns. PLOS regrets that this issue was not identified prior to the article’s publication.

The *PLOS One* Editors retract this article in light of the above concerns which call into question the validity and reliability of the reported results.

MDP did not agree with the retraction. LS, JF, KN, ELD, MP, MF, BK, and VA either did not respond directly or could not be reached.
